# Association of abdominal aortic aneurysm diameter with insulin resistance index

**DOI:** 10.11613/BM.2018.030702

**Published:** 2018-10-15

**Authors:** Fabien Lareyre, Claudine Moratal, Elamine Zereg, Joseph Carboni, Patricia Panaïa-Ferrari, Pascale Bayer, Elixène Jean-Baptiste, Réda Hassen-Khodja, Giulia Chinetti, Juliette Raffort

**Affiliations:** 1 Department of Vascular Surgery, University Hospital of Nice, Nice, France; 2 Université Côte d’Azur, CHU, Inserm, C3M, Nice, France; 3 Department of Clinical Biochemistry, University Hospital of Nice, Nice, France

**Keywords:** diabetes mellitus, abdominal aortic aneurysm, C-peptide, insulin resistance, fructosamine

## Abstract

**Introduction:**

Epidemiological studies have highlighted a negative association between diabetes and abdominal aortic aneurysm (AAA). The aim of this study was to investigate the association between insulin resistance and AAA size.

**Materials and methods:**

This prospective cross sectional monocentric study analysed fasting blood samples from 55 patients with AAA eligible for surgical repair. They were divided into 2 groups according to the median AAA diameter: diameter < 50 mm (N = 28) and diameter > 50 mm (N = 27). The median ages were respectively 73 years (62 - 79) and 72 years (67 - 81). Glucose and fructosamine concentrations were determined by spectrophotometry; insulin and C-peptide using chemiluminescent technology. Homeostasis model assessment 2 calculator was used to estimate insulin resistance index (HOMA2 IR).

**Results:**

There was no significant difference for fasting glucose concentration between the groups (6.1 *vs*. 5.9 mmol/L, P = 0.825). C-peptide and insulin concentrations, as well as HOMA2 IR index were significantly higher in patients with AAA > 50 mm (0.82 *vs*. 0.54 nmol/L, P = 0.012; 9 *vs*. 5 mU/L, P = 0.019 and 1.72 *vs*. 1.26, P = 0.028, respectively). No linear correlation was identified between AAA diameter and HOMA2 IR. Fructosamine concentration was lower in patients with AAA > 50 mm (225.5 *vs*. 251 μmol/L, P = 0.005) and negatively correlated with AAA diameter (r = - 0.54, P < 0.001).

**Conclusion:**

This study evidenced an association between AAA diameter and insulin resistance. Further studies are required to determine a causal link between insulin resistance and AAA development.

## Introduction

Abdominal aortic aneurysm (AAA), generally defined as a focal dilatation of the aorta superior to 30 mm in diameter, represents a life-threatening disease (*1*). It is estimated to be the tenth commonest cause of mortality, as a result of severe complications such as aortic rupture (*2*). Despite advances in the management of patients, specific pharmacological approaches to treat and limit aneurysm expansion are still lacking and the only curative therapeutic option relies on surgical interventions including open and endovascular surgery (*1*, *2*). The decision to treat patients relies on the balance between the operative risks and the risks of progression and rupture. Symptomatic aneurysms, which are often manifested by abdominal or back pain, or rupture, should be promptly treated (*1*). For asymptomatic patients, there is general agreement that small aneurysms (< 40 mm in diameter) and at low risk of rupture should be monitored, whereas bigger aneurysms (> 54 mm) or at high risk of rupture should be repaired (*1*).

Abdominal aortic aneurysm is most often associated with atherosclerosis and corresponding cardiovascular risk factors including age, male sex, smoking, arterial hypertension and dyslipidaemia (*3*, *4*). Abdominal aortic aneurysm formation results from a complex process involving mainly extra-cellular matrix (ECM) remodelling, infiltration of inflammatory cells within the aortic wall as well as impairment of vascular smooth muscle cell homeostasis and increased oxidative stress (*3*). Intriguingly, while diabetes mellitus represents a major cardiovascular risk factor, epidemiological studies have pointed out a negative association between diabetes and AAA (*5*-*12*). Both prevalence and incidence of AAA were found to be lower in diabetic patients compared to non-diabetics (*6*-*10*, *13*). In addition, several reports have shown that diabetic patients develop smaller aneurysm and have lower growth rates of AAA (*6*, *8*, *13*-*17*). At last, a negative association was found between diabetes and AAA rupture (*18*). These results suggest a protective effect of diabetes on AAA formation and the understanding of the mechanisms involved could bring innovative therapeutic strategies for the patients.

The pathophysiological mechanisms underlying the protective effect of diabetes on AAA occurrence and development are not totally understood and may be multifactorial through effects on ECM remodelling, inflammation, vascular smooth muscle cell homeostasis, neoangiogenesis, and thrombus formation (*6*, *19*). In addition, the use of antidiabetic drugs has also been paradoxically identified as a protective factor against AAA formation (*20*, *21*).

Type 2 diabetes, which accounts for 90% of diabetic patients, is characterized by a chronic hyperglycaemia resulting from insulin resistance associated with defects in insulin secretion. Several studies have previously addressed the link between hyperglycaemia on AAA development. An inverse correlation was found between fasting blood glucose and glycated haemoglobin A1c (reflecting long-term glucose concentrations) with AAA diameter (*14*, *16*). While the link between blood glucose concentration and AAA development is well described, the association between AAA and insulin resistance has never been explored. We previously published a pilot study which compared plasma inflammatory profile between diabetic and non-diabetic patients with AAA (*19*). However, we did not address the association between insulin resistance and AAA independently of diabetic status. Insulin resistance impairs vascular function and play roles in mechanisms potentially relevant for AAA pathogenesis. Insulin resistance is associated with increased oxidative stress and inflammation and can impact on vascular smooth muscle cells survival (*22*-*25*). We hypothesized that unlike chronic hyperglycaemia, insulin resistance may be positively associated with AAA development. Based on our previously published cohort, the aim of this study was to investigate the association between insulin resistance and AAA size.

## Material and methods

### Study design and subjects

This prospective monocentric cross-sectional study included patients with AAA eligible to surgical repair in the Department of Vascular Surgery at the University Hospital of Nice from January 2016 to February 2017. This study involves a previously published clinical cohort (*19*). It was conducted in conformity with the Declaration of Helsinki and was approved by our institutional local ethics committee (Comité de Protection des Personnes Sud Méditerranée V). All enrolled patients gave informed written consent.

Inclusion criteria were patients over 18 years with AAA eligible to aortic surgical repair. The diagnosis of AAA was defined as a focal dilatation of the abdominal aorta with a diameter superior to 30 mm, in accordance with the current guidelines of vascular surgery (*1*). Abdominal aortic aneurysm diameter was measured on injected CT-scan by trained vascular surgeons. Eligibility to aortic surgical repair was discussed by a multidisciplinary team composed of vascular surgeons and anaesthetists based on the balance between the operative risk and the risk of progression and rupture (*1*). The aetiologies of AAA were evaluated based on patient’s history, clinical presentation, results of biological and imaging investigations. Patients who had a declared history of systemic inflammatory disease (connective tissue disease or vasculitis) or a genetic disorder potentially leading to AAA (Marfan, Ehlers Danlos or Loeys Dietz syndrome) were excluded. Patients who had a mycotic aneurysm were excluded based on the results of biological and imaging investigations.

At the time of inclusion, clinical pre-operative characteristics were collected including age, sex, the presence of diabetes, arterial hypertension, dyslipidaemia and smoking habits, based on patient’s declaration, medical records and treatments. The height and the weight were measured and the body mass index was calculated by dividing the weight (kg) by the square of the body height (m^2^). The patient treatments were recorded based on medical prescriptions. Data were collected using electronic or manuscript medical records, as well as by a computer software program named Clinicom® (InterSystems Corporation, Cambridge, USA). Imaging data were recorded and extracted from the Picture Archiving and Communication System (PACS®) multiple modalities software. Post-processing analysis was performed using the Aquarius® workstation (TeraRecon Inc., San Mateo, USA).

In total, 55 patients were included. To investigate the link between glycaemic parameters, insulin resistance and AAA size, the study population was divided into 2 subgroups (N = 28 and N = 27, respectively) based on the median value of the AAA diameter. The median value of AAA diameter was 50 mm (interquartile range: 46 - 56). Patients with AAA < 50 mm had a median age of 73 years (62 - 79) and patients with AAA > 50 mm had a median age of 72 years (67 - 81).

### Blood sampling

Blood samples were obtained after a peripheral vein puncture from the antecubital vein after 8 to 12 hours fasting the day before the surgical intervention. Blood was collected into 5 mL BD Vacutainer® tubes (Becton Dickinson and Company, Le Pont de Claix, France). Blood was collected in two different vacutainer tubes. Tube with serum separator and clot activator for determination of insulin, C-peptide and fructosamine was used, and for plasma glucose measurement tube coated with sodium fluoride was used. Blood was collected in the Department of Vascular Surgery and immediately sent to the Department of Clinical Biochemistry. Samples were centrifuged at 20 °C for 10 minutes at 3000xg within 2 hours after collection and sera were immediately analysed. Two aliquots of remaining sera (200 µL each) were then stored at - 80 °C for back-up.

### Methods

All the analyses were performed in the Clinical Chemistry Laboratory at the University Hospital of Nice using standard methods. All the analyses were certified by the National French Committee of Accreditation. Calibration was done before quality controls according to the manufacturer’s instructions. For all assays, quality controls fell within limits defined by the manufacturer and current guidelines (*26*, *27*). Glucose and fructosamine concentrations were determined by spectrophotometry (Cobas 8000, Roche Diagnostics, Meylan, France). Calibrators and controls were obtained from Roche (Roche Diagnostics, Meylan, France); blank and Calibrator for Automated Systems (10.8 mmol/L) for determination of glucose; blank and Precimat Fructosamine (400 µmol/L) for the measure of fructosamine. For glucose determination, two quality controls were used: Precicontrol ClinChem Multi 1 (5.6 mmol/L) and Precicontrol ClinChem Multi 2 (13.4 mmol/L). The inter-assay coefficients of variation (CVs) were 1.03% and 1.12%, respectively. For fructosamine determination controls used were: Precinorm Fructosamine (280 µmol/L) and Precipath Fructosamine (540 µmol/L). The inter-assay CVs were 1.9% and 1.6%, respectively.

Insulin and C-peptide were measured with a sandwich immunoassay using direct chemiluminescent technology (Centaur XP, Siemens Healthineers GmbH, Erlangen, Germany). Calibrators were obtained from Siemens Healthineers. For insulin determination, three quality controls from Biorad Clinical Diagnostics (Marnes-la-Coquette, France) were used: Liquichek Immunoassay Plus Quality Control (19, 78 and 192 mU/L). The inter-assay CVs were 5.0, 4.8 and 5.2%, respectively. For C-peptide determination, three quality controls from Biorad Clinical Diagnostics (Marnes-la-Coquette, France) were used: Liquichek Specialty Quality Control (0.416, 1.630 and 4.650 nmol/L). The inter-assay CVs were 4.4, 5.3 and 4.6%, respectively.

Fasting glucose concentrations were interpreted according to the criteria defined by the American Diabetes Association (*28*). Diabetes was defined as fasting glucose concentration > 7.0 mmol/L. Impaired fasting glucose concentration corresponded to glucose concentration between 5.6 and 6.9 mmol/L.

Homeostasis model assessment 2 (HOMA2) calculator was used to estimate steady state beta cell function (HOMA 2%B), insulin sensitivity (HOMA2%S) and insulin resistance index (HOMA2 IR) from fasting glucose and C-peptide concentration (*29*). As stated by the “Diabetes Trials Unit from the Oxford Centre for Diabetes”, no absolute defined threshold for normal ranges for HOMA values exist and HOMA indices were directly compared between the two study groups.

### Statistical analysis

Categorical data were expressed as the number of patients and ratios, and continuous variables were expressed as the median with interquartile ranges. Given the low number of patients, group differences were compared using non-parametric Mann-Whitney test. Fisher’s exact test was used for categorical data. Correlations were determined by non-parametric Spearman’s correlation coefficient. A P value < 0.05 was considered as significant. Statistical analyses were performed using GraphPad Prism® software (version 7.00, La Jolla California USA).

## Results

The general characteristics of patients enrolled in the study are presented in Table 1. No significant difference was observed for the sex ratio between patients with AAA < 50 mm and those with AAA > 50 mm (male: 25/28 and 23/27, P = 0.705). The median body mass index did not significantly differ (24.5 *vs* 24.5, P = 0.145). There was no significant difference regarding the proportion of patients with arterial hypertension, dyslipidaemia or smoking habits between the 2 groups. No difference was observed regarding the treatments including the use of statins, antiplatelets, antihypertensives or oral anti-diabetic drugs between the 2 groups.

**Table 1:** Characteristics of patients enrolled in the study

**Characteristics**	**Patients with AAA** **< 50 mm** **(N = 28)**	**Patients with AAA** **> 50 mm** **(N = 27)**	**P**
Age (years)	73 (62 - 79)	72 (67 - 81)	0.363
Male sex, N (proportion)	25 (0.89)	23 (0.85)	0.705
BMI (kg/m^2^)	24.5 (26.9 - 30.5)	24.6 (30 - 34.5)	0.145
Type 2 diabetes, N (proportion)	6 (0.21)	5 (0.19)	0.999
Arterial hypertension, N (proportion)	16 (0.57)	18 (0.67)	0.582
Dyslipidaemia, N (proportion)	11 (0.39)	5 (0.19)	0.138
Smoking, N (proportion)	21 (0.75)	20 (0.74)	0.999
Use of statins, N (proportion)	18 (0.64)	14 (0.52)	0.418
Use of antiplatelets, N (proportion)	19 (0.68)	21 (0.78)	0.547
Use of antihypertensive, N (proportion)	15 (0.54)	19 (0.70)	0.270
Use of oral anti-diabetics, N (proportion)	6 (0.21)	5 (0.19)	0.999
AAA diameter (mm)	47 (37 - 50)	56 (53 - 67)	< 0.001
Fasting glucose (mmol/L)	5.9 (4.8 - 7.2)	6.1 (5 - 7.5)	0.825
C-peptide (nmol/L)	0.54 (0.38 - 1.02)	0.82 (0.68 - 1.13)	0.012
Insulin (mU/L)	5 (3 - 8)	9 (5 - 13)	0.019
Fructosamine (μmol/L)	251 (225 - 275)	226 (212 - 247)	0.005
HOMA2%B	68 (49 - 136)	97 (55 - 137)	0.491
HOMA2%S	79 (47 - 115)	58 (36 - 70)	0.029
HOMA2 IR	1.3 (0.9 - 2.1)	1.7 (1.4 - 2.8)	0.028
AAA - abdominal aortic aneurysm. BMI - body mass index. HOMA2%B - steady state beta cell function estimated according to HOMA2 calculator. HOMA2%S - insulin sensitivity estimated according to HOMA2 calculator. HOMA2 IR - insulin resistance estimated according to HOMA2 calculator. Values are median (interquartile range) or N (proportion). P value < 0.05 was considered as significant.			

Fasting glucose concentration did not significantly differ between the 2 groups (6.1 *vs* 5.9 mmol/L, P = 0.825). There was no significant difference regarding the proportion of patients with a declared history of type 2 diabetes between the 2 groups (6/28 *vs* 5/27, P = 0.999). When analysing fasting glucose concentration, further 5/28 patients with AAA < 50 mm and 6/27 patients with AAA > 50 mm had a glucose concentration > 7 mmol/L, which defines diabetic state, but were unaware of their diagnosis. Eight out of 28 patients with AAA < 50 mm and 7/27 patients with AAA > 50 mm had a glucose concentration between 5.6 and 6 mmol/L, which corresponds to impaired fasting glucose concentration defining prediabetic state.

C-peptide and insulin concentrations were significantly higher in patients with AAA > 50 mm compared to those with AAA < 50mm (0.82 *vs* 0.54 nmol/L, P = 0.012 and 9 *vs* 5 mU/L, P = 0.019, respectively). Insulin resistance index was also significantly higher in patients with AAA > 50 mm (HOMA2 IR: 1.72 *vs* 1.26, P = 0.028). There was no significant difference for the steady state beta cell function (HOMA2%B) between the 2 groups (97.3 *vs* 68.3%, P = 0.491). Fructosamine concentration was significantly lower in patients with AAA > 50 mm (225.5 *vs* 251 μmol/L, P = 0.005).

We further investigated the potential correlation between glycaemic parameters and AAA size (Table 2). In our study, glucose, C-peptide, insulin concentrations and insulin resistance (HOMA IR) did not directly correlate with AAA diameter, as revealed by Pearson’s correlation coefficient closed to zero. However, fructosamine concentration negatively correlated with AAA size (r = - 0.54, P < 0.001).

**Table 2:** Correlation analyses between glycaemic parameters and AAA diameter

**Glycaemic parameters**	**r**	**P**
Fasting glucose (mmol/L)	0.04	0.797
C-peptide (nmol/L)	0.15	0.327
Insulin (mU/L)	0.13	0.385
Fructosamine (μmol/L)	- 0.54	< 0.001
HOMA2%B	- 0.06	0.665
HOMA2%S	- 0.09	0.544
HOMA2 IR	0.09	0.538
r - Spearman’s correlation coefficient. HOMA2%B - steady state beta cell function estimated according to HOMA2 calculator. HOMA2%S - insulin sensitivity estimated according to HOMA2 calculator. HOMA2 IR - insulin resistance estimated according to HOMA2 calculator. P value < 0.05 was considered as significant.		

## Discussion

Most of the studies published so far compared features between diabetic and non-diabetic patients with AAA (*19*, *30*-*34*). To the best of our knowledge, this is the first study addressing the association between insulin resistance and AAA. We found that patients with AAA > 50 mm had significantly higher C-peptide and insulin concentrations compared to patients with AAA < 50 mm, despite no difference in fasting blood glucose. These results are in accordance with a previous published study reporting a positive correlation between C-peptide concentration and AAA diameter (*35*). In addition, we found that patients with AAA > 50 mm had significantly higher insulin resistance index as estimated by HOMA2 calculator, which evidenced an association between AAA diameter and insulin resistance index. However, no direct correlation was found between AAA diameter and HOMA2 IR, suggesting that the link between both factors may be more complex than previously anticipated. The HOMA2 model estimates insulin resistance according to fasting glucose concentration and insulin or C-peptide concentrations and takes into account variations in hepatic and peripheral glucose resistance. There is currently no absolute established threshold to define normal ranges for HOMA values as it depends on the specific assays used for glucose, insulin and C-peptide measures as well as subject ethnicity and gender (*29*, *36*, *37*). Hence, it would be worth further to confirm the association between insulin resistance and AAA size using other established and innovative biomarkers of insulin resistance (*38*).

While this study evidenced an association between AAA diameter and insulin resistance index, it cannot be given any conclusion whether this association is protective or not against AAA formation. However, clinical and fundamental research performed so far suggest that insulin resistance may favour AAA initiation and progression. Epidemiological studies revealed that the use of insulin sensitizers including biguanides (metformin) and thiazolidinediones (rosiglitazone, pioglitazone) was associated with a lower risk of developing aneurysm (*20*). To go further in the cellular pathways involved, experimental studies have been performed in chemically induced aneurysm animal models (*39*, *40*). Elastase model requires the application or the perfusion of pancreatic porcine elastase on the infrarenal abdominal aorta in C57Bl6j mice and leads to AAA formation within 14 days. The angiotensin II model relies on a continuous subcutaneous angiotensin II infusion in C57Bl6j or apoE-/- mice inducing AAA usually located in the suprarenal aorta within 28 days. Interestingly, the administration of metformin reduced the development of elastase-induced AAA, an effect associated with a preservation of vascular smooth muscle cells and aortic medial elastin and a decreased inflammatory cell infiltration (*41*).

Similarly, administration of thiazolidinediones to apoE-/- mice perfused with angiotensin II reduced aortic dilatation as well as macrophage infiltration in aneurysmal tissues (*42*).

The link between glycaemic parameters and AAA development has been addressed by several studies. Some investigators identified an inverse correlation between AAA diameter and fasting blood glucose concentration (*14*). In our study, we did not reproduce this result. This could be, at least, partly explained by the small size of our cohort, which may have limited the statistical power of the analysis. Besides, blood glucose concentration is highly variable throughout the day and does not reflect long-term glucose homeostasis (*28*). By contrast, fructosamine has the advantage to reflect the average glycaemic status over the preceding 2 to 3-week period and its measurement is quick, cheap and fairly free of analytic interferences (*43*). Interestingly, a negative correlation between fructosamine concentrations and AAA diameter was observed, as revealed by a Spearman’s coefficient correlation of - 0.54, P < 0.001. To the best of our knowledge, this is the first report that investigated the link between fructosamine and AAA. Other studies have explored the association between HbA1c, which reflects the glycaemic status over the past 3 months, and identified an inverse association between the growth rate of AAA and the level of HbA1c (*16*, *28*). Taken together, these results underline the negative association between long-term high plasma glucose concentrations and AAA diameter.

At last, we measured fasting glucose concentration. Even though the aim of this study was not to determine diabetic states, we found that some patients who did not have a declared history of diabetes had impaired fasting glucose concentration or glycaemia > 7 mmol/L. These results suggest that many patients are unaware that they are diabetic or pre-diabetic and point to an underestimation of diabetes in patients with AAA. This is in accordance with another study which found that almost half of patients with AAA were unaware that they were diabetics according to HbA1c and glucose concentration obtained after an oral glucose tolerance test (*44*). These findings underline the real need in practice to improve the detection of diabetes in patients with AAA.

This study presents some limitations. First, this was a single-centre study involving a small number of patients. This could have potentially limited the statistical power of the analyses. Second, even if the Homeostasis Modell Assessment is a well- established tool to evaluate insulin resistance, absolute established threshold to define normal ranges are lacking (*28*). The normal reference ranges for HOMA-IR, HOMA %B and HOMA %S are impacted by ethnic group and needs to be determined for every community (*30*, *31*). The determination of normal ranges specifically adapted to our cohort would have required to determine insulin resistance indices in a test group and to compare it with analytes reflecting insulin resistance symptoms and pathology. This study was not designed to address it, which represents a limitation. Nevertheless, this study allowed a relative comparison of insulin resistance indices between patients with AAA > 50 mm and those with AAA < 50 mm. It would be worth to extend this work on larger cohorts and to use combined biomarkers of insulin resistance to precisely characterize its severity and its association with AAA.

This study is of interest as to the best of our knowledge, this is the first to investigate the association between AAA size and insulin resistance. This may serve as a basis to perform clinical studies on larger cohorts as well as experimental researches on animal models to explore the molecular and cellular pathways relaying the effect of insulin resistance on AAA formation. At long term, this could lead to develop new therapeutic strategies for patients with AAA.

In conclusion, to the best of our knowledge, this is the first clinical study addressing the association between insulin resistance and AAA size. This study evidenced an association between AAA diameter and insulin resistance index. However, insulin resistance did not directly correlate with AAA diameter, pointing to a complex association between these two factors. Further clinical and fundamental studies are required to establish a causal link between insulin resistance and AAA development and determine the molecular and cellular pathways underlying this association.
